# Counter-current chromatography for the separation of terpenoids: a comprehensive review with respect to the solvent systems employed

**DOI:** 10.1007/s11101-014-9348-2

**Published:** 2014-03-25

**Authors:** Krystyna Skalicka-Woźniak, Ian Garrard

**Affiliations:** 1 Department of Pharmacognosy with Medicinal Plant Unit, Medical University of Lublin, 1 Chodzki Str., 20-093 Lublin, Poland; 2 Advanced Bioprocessing Centre, Brunel Institute for Bioengineering, Brunel University, Uxbridge, UB8 3PH UK

**Keywords:** Counter-current chromatography, Terpenoids, Terpenes, Natural products, Separation, Purification

## Abstract

Natural products extracts are commonly highly complex mixtures of active compounds and consequently their purification becomes a particularly challenging task. The development of a purification protocol to extract a single active component from the many hundreds that are often present in the mixture is something that can take months or even years to achieve, thus it is important for the natural product chemist to have, at their disposal, a broad range of diverse purification techniques. Counter-current chromatography (CCC) is one such separation technique utilising two immiscible phases, one as the stationary phase (retained in a spinning coil by centrifugal forces) and the second as the mobile phase. The method benefits from a number of advantages when compared with the more traditional liquid–solid separation methods, such as no irreversible adsorption, total recovery of the injected sample, minimal tailing of peaks, low risk of sample denaturation, the ability to accept particulates, and a low solvent consumption. The selection of an appropriate two-phase solvent system is critical to the running of CCC since this is both the mobile and the stationary phase of the system. However, this is also by far the most time consuming aspect of the technique and the one that most inhibits its general take-up. In recent years, numerous natural product purifications have been published using CCC from almost every country across the globe. Many of these papers are devoted to terpenoids—one of the most diverse groups. Naturally occurring terpenoids provide opportunities to discover new drugs but many of them are available at very low levels in nature and a huge number of them still remain unexplored. The collective knowledge on performing successful CCC separations of terpenoids has been gathered and reviewed by the authors, in order to create a comprehensive document that will be of great assistance in performing future purifications.

## Introduction

Terpenoids, also referred to as terpenes, are one of the largest and the most diverse group of natural products accounting for more than 40,000 individual compounds, with several new compounds being discovered every year.

**Fig. 1:**
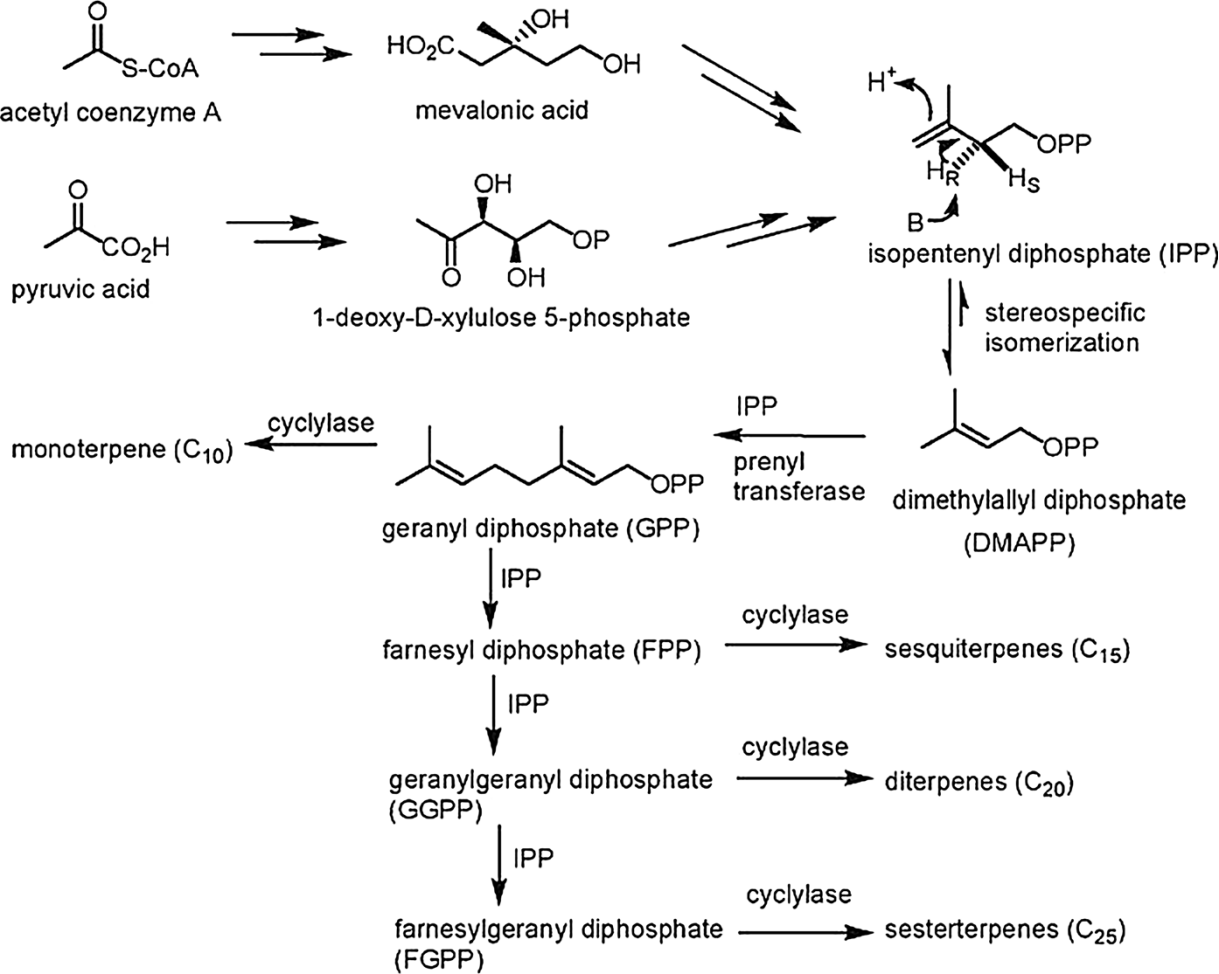
Biosynthetic pathways of terpenes (Wang et al. 2005)

They are synthesized from only two five-carbon isomers: isopentenyl diphosphate (IPP) and dimethylallyl diphosphate (DMAPP). Two biosynthetic routes have been characterized: the classical acetate mevalonate pathway (described in 1967) and the triose phosphate-utilizing non-mevalonate pathway characterized in 2002. Starting from the universal precursors IPP and DMAPP, thousands of enzymes are involved in the biosynthetic pathways for terpenoid chain elongation, cyclization, and functionalization of hydrocarbon chains. The active isoprene unit (IPP) is repetitively added to DMAPP or a prenyl diphosphate in sequential head-to-tail condensations catalyzed by the prenyltrans-ferases. Through consecutive condensations a prenyltransferase can synthesize a variety of products with fixed lengths and stereochemistry (Fig. 1) (Wang et al. 2005; Ajikumar et al. 2008). Based on the number of the building blocks, terpenoids are commonly classified into hemi-, mono-, sesqui-, di-, ses-, tri- and tetraterpenoids (carotenoids) having 1, 2, 3, 4, 5, 6 and 8 isoprenoid residues respectively, and polyterpenes consisting of long chains of many isoprene units (Koch et al. 2008).

Terpenoids display a wide range of biological activities. Monoterpenes and sesquiterpenes are the main constituents of essential oils and share responsibility for important properties like antibacterial, antiviral, antioxidant etc. Triterpene saponins—ginsenosides—significantly reduce the production of beta-amyloid which accumulates in the brain of patients with Alzheimer’s disease and play a critical role in pathology by inducing neuronal death. Ginkgolides (cyclic diterpenes of labdane type commonly isolated from *Ginkgo biloba*) protect neuronal cells from synaptic damage (Yoo and Park 2012). However the antimalarial drug Artemisininn and the anticancer drug paclitaxel (TaxolR) are two renowned terpene-based drugs with established medical applications. Artemisinin, earlier known as *Qinghaosu*, is a phytoconstituent isolated from *Artemisia annua* L. and can be described as a compound which possess antimalarial activity. Clinical studies with patients infected with *Plasmodium vivax* or *P. falciparum* demonstrated that artemisinin could kill the malarial parasite very quickly at the schizont stage of the parasite’s life cycle (i.e. when it infects the human red blood cell) with no obvious side effects. The molecule has a completely new antimalarial prototype structure with an endoperoxide moiety, which is necessary for activity. Since artemisinin itself has poor bioavailability limiting its effectiveness, several semisynthetic derivatives of artemisinin have been developed (Medhi et al. 2009; Brown 2010). Taxol, a plant diterpenoid widely used as a chemotherapeutic drug against several types of cancer, is known to interact with a specific site of β-tubulin—it binds to microtubules and inhibits their disassembly. Cells treated with taxol are arrested in mitosis and eventually undergo death by apoptosis. This very important activity is strongly depended on its unusual structure. It was shown that the side chain at position C-13 and the taxane ring system are essential for this activity (Xiang et al. 2009).

The purification of natural products is a complex process requiring a comprehensive range of techniques. CCC offers the natural product scientist a different mode of operation to conventional processes. Invented in the mid 1960’s (Ito et al. 1966), to many scientists it is still known as it was back then—a technique that is slow, with separations measuring in hours or days. It was also unreliable as instruments frequently broke down and furthermore had poor capacity with injection amounts measured in tens of milligrams. There was also no opportunity for scale up as the factors required to scale up were poorly understood. However, the technique has been substantially developed since those early days. Advances in engineering and the understanding of the processes involved, particularly in the past 10 years, have created instruments that are fast, robust, permit very high injection loadings and, significantly for the natural products industry, can be rapidly scaled from analytical to pilot level (Sutherland and Fisher 2004). The new generation of coil planet centrifuges operate at higher “*g*” fields than conventional instruments, enabling higher flow rates to be used so that separation times are measured in minutes rather than hours at the same resolution (Yuan et al. 2008). With a number of important advantages over both solid phase chromatographic techniques and current liquid–liquid extraction techniques, modern high capacity counter-current chromatography is a worthy inclusion in the array of techniques required for natural product purifications.

## Theoretical background of CCC

**Fig. 2:**
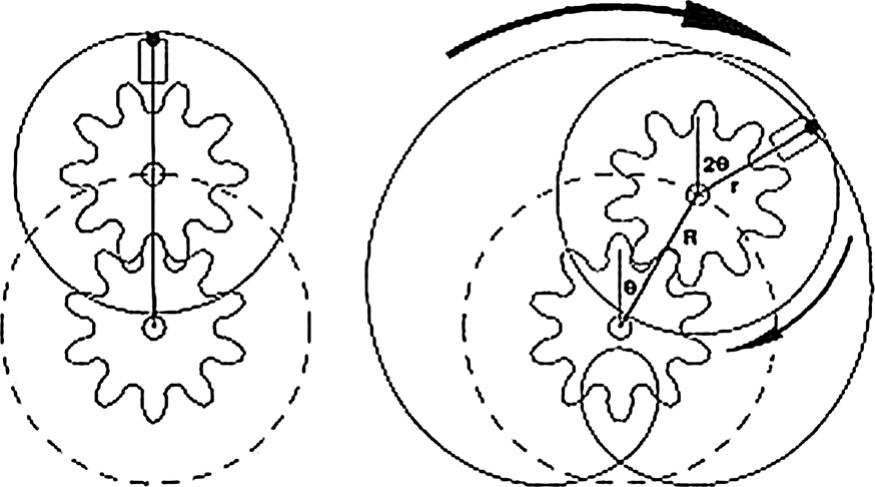
Motion of the bobbin in the CCC centrifuge (Sutherland et al. 1998)

In a counter-current chromatography centrifuge, tubing is wound on a drum which is centrifugally rotated in planetary motion (the holder rotates about its own axis while revolving around the centrifuge axis at the same angular velocity in the same direction) (Fig. 2). A two phase solvent system is introduced into this coil. Although a simple solvent system might consist of hexane and water, a more likely system for a purification would consist of hexane, ethyl acetate, methanol and water or, for biomolecules sensitive to organic solvents, an aqueous two phase system such as aqueous PEG1000 and potassium phosphate salt solution. With the two phase solvent system inside, as the coil travels through its planetary motion cycle, zones of mixing and settling travel along the phases coincident with the low and high accelerations caused by the epicyclic motion of the coil. The mixing zones are coincident with low accelerations and take the form of wave mixing, equivalent to the “swish-swosh” motion that occurs when a tube of liquids is tilted from side to side. The settling zones are coincident with high accelerations and take the form of a smooth interfacial area. There is one mixing and one settling zone per coil loop per revolution. A typical modern analytical CCC instrument may have 40 loops and spin at 2,000 rpm. It will therefore experience 4.8 million partitioning steps per hour (40 × 2,000 × 60). Similarly, a typical preparative instrument may have 30 loops and spin at 1,200 rpm, giving 2.2 million partitioning steps per hour.

**Fig. 3:**
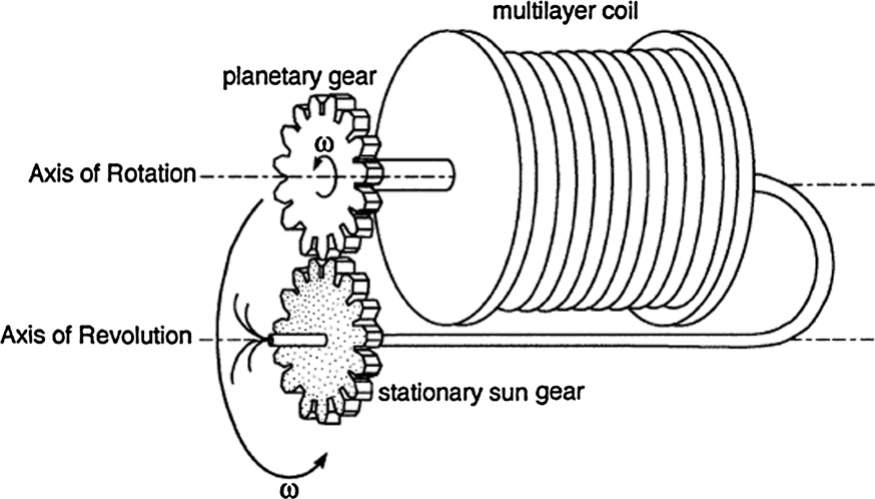
Type-J planetary motion of a multilayer coil separation column presenting that the column holder rotates about its own axis and revolves around the centrifuge axis at the same angular velocity (*ω*) in the same direction (Ito 2005)

Counter-current chromatography can be achieved not only in the above mentioned hydrodynamic CCC created by two axis of rotation, but also as hydrostatic CCC, typically represented by centrifugal partition chromatography (CPC). This is a single-axis instrument, which has a series of chambers machined circumferentially around a rotor. Rotation of the rotor produces a uniform g-field, which retains the stationary phase in each chamber, while the mobile phase is flowed though in a cascading manner. A CPC instrument requires rotating seals for the mobile phase flow, whereas the hydrodynamic CCC instruments do not. This is because the rotation of the coil about its own axis unwinds the twist produced by its motion around the sun gear, and thus there is no twisting of the flow tubes linking the coil to the pump and the detector (Fig. 3).

## Solvent selection process

In the past, selection of a suitable two phase solvent system involved a considerable amount of experience and know-how. With aqueous-organic phase systems coming from up to six or more different solvents mixed together, the possibilities were almost limitless. A typical mid-polarity selection table is shown in Table 1 (Garrard 2005). This is a modified version of the table produced by Oka et al. (1991) and runs from moderately polar (System No1: butanol–water) to moderately nonpolar (System No28: heptane–methanol). The italicized systems in the table can be used to rapidly screen the whole table first, allowing the operator to focus into the correct area of interest. Being all multiples of 0.5 ml when 4 ml solvent system is made, these particular systems are quick to make up and test. It is also possible to create the solvent systems on a micro-scale in 96 well plates if the crude sample is in short supply.

**Table 1:** Table for selecting a suitable moderately polar two-phase solvent system, graded from polar (No1) to nonpolar (No28)

No	Heptane	EtOAc	MeOH	Butanol	Water
* 1*	*0*	*0*	*0*	*2*	*2*
2	0	0.4	0	1.6	2
3	0	0.8	0	1.2	2
4	0	1.2	0	0.8	2
5	0	1.6	0	0.4	2
* 6*	*0*	*2*	*0*	*0*	*2*
7	0.1	1.9	0.1	0	1.9
8	0.2	1.8	0.2	0	1.8
9	0.29	1.71	0.29	0	1.71
10	0.33	1.67	0.33	0	1.67
11	0.4	1.6	0.4	0	1.6
* 12*	*0.5*	*1.5*	*0.5*	*0*	*1.5*
13	0.57	1.43	0.57	0	1.43
14	0.67	1.33	0.67	0	1.33
15	0.8	1.2	0.8	0	1.2
16	0.91	1.09	0.91	0	1.09
* 17*	*1*	*1*	*1*	*0*	*1*
18	1.09	0.91	1.09	0	0.91
19	1.2	0.8	1.2	0	0.8
20	1.33	0.67	1.33	0	0.67
21	1.43	0.57	1.43	0	0.57
* 22*	*1.5*	*0.5*	*1.5*	*0*	*0.5*
23	1.6	0.4	1.6	0	0.4
24	1.67	0.33	1.67	0	0.33
25	1.71	0.29	1.71	0	0.29
26	1.8	0.2	1.8	0	0.2
27	1.9	0.1	1.9	0	0.1
*28*	*2*	*0*	*2*	*0*	*0*

Quantities (in ml) required to make 4 ml of system using a liquid-handling robot. EtOAc = ethyl acetate, MeOH = methanol. Hexane may be used instead of heptane (Garrard 2005)

In order to achieve an efficient resolution of the target compounds, the *K* values, which express the solute concentration in the stationary phase divided by that in the mobile phase, should be calculated. The partition coefficient (*K*) should lie within the approximate range 0.5 < *K* < 2.0. A smaller *K* value results in a loss of peak resolution, whilst a larger value produces excessive band broadening.

## Solvent systems employed in CCC for terpenoid purification

In order to fully assess the use of two phase systems in CCC of terpenoids, approximately 3,500 scientific papers were studied, published in the last 30 years, that related to all aspects of CCC or CPC. Papers that contained an application example of terpenoids, i.e. a purification performed by CCC or CPC were noted, together with the compounds purified and the solvent system used for the purification. Papers which gave examples of separations reported elsewhere were ignored, as were all symposium abstracts. Only papers which gave specific details of the solvent system and solute were recorded and only natural product secondary metabolites were noted e.g. no synthetic compounds, dyes or chemicals.

In total therefore, 150 solvent systems were listed in Table 2 together with the corresponding solutes that they separated. Some of the solvent systems corresponded to more than one solute, and some of the solutes corresponded to more than one solvent system, but if the same solute and the same solvent system were listed, this was simply a duplicate entry and was therefore removed.

**Table 2:** Solvent systems in CCC for terpenoids separations

Classes of compounds	Purified compounds	References	Type of apparatus/solvent system
Monoterpenoids	Paeoniflorin Albiflorin	Huang et al. (2013)	CCC/ethyl acetate–*n*-butanol–water (3:2.5:5)
	Rosiridin Geranyl 1-*O*-α-l-arabinopyranosyl-(1→6)-β-d-glucopyranoside	Mudge et al. (2013)	CCC/ethyl acetate–butanol–water (3:2:5)
	(*S*)-3,7-Dimethyl-5-octene-1,7-diol	Knapp et al. (1998)	CCC/chloroform–methanol–water (7:13:8)
	Thymol Carvacrol	Puertas Mejia et al. (2002)	CCC/*n*-hexane–*tert*-butylmethyl ether–acetonitrile (1:0.1:1)
	Eugenol	Geng et al. (2007)	CCC/n-hexane–ethyl acetate–methanol–water (1:0.5:1:0.5)
	Chavibetol Methyleugenol	dos Santos et al. (2009)	CCC/*n*-hexane–n-butanol–methanol–water (12:4:4:3)
	α-Cyperone	Shi et al. (2009)	CCC/*n*-hexane–ethyl acetate–methanol–water (1:0.2:1.1:0.2)
	1,8-Cineole	Dang et al. (2010)	CPC/petroleum ether–acetonitrile–acetone (4:3:1)
	Paeoniflorin	Chen et al. (2004)	CCC/n-butanol–ethyl acetate–water (1:4:5)
	Cuminaldehyde *p*-Menta-1,4-dien-7-al	Chen et al. (2011)	CCC/n-hexane–methanol–water (5:4:1)
	Linalol Terpinen-4-ol α-Terpineol	Skalicka-Woźniak et al. (2013)	CCC/heptane–ethyl acetate–methanol–water (5:2:5:2)
	Anethole Foeniculin	Skalicka-Woźniak et al. (2013)	CCC/heptane–methanol (1:1)
Sesquiterpenoids	Parthenolide 11,13-Dihydroparthenolide Anhydroverlotorin 3β-Hydroxycostunolide Costunolide diepoxide 3-Hydroxyparthenolide Artemorin Santamarine Reynosin Artecanin Tanaparthin-β-peroxide	Fischedick et al. (2012)	CPC/heptane-ethyl acetate–methanol–water (1:1:1:1)
	Artemisinin	Acton et al. (1986)	CCC/*iso*-octane-ethyl acetate–methanol–water (7:3:6:4)
	Costunolide Dehydrocostuslactone	Li et al. (2005)	CCC/light petroleum–methanol–water (5:6.5:3.5)
	Lactucopicrin	Wu et al. (2007)	CCC/*n*-hexane–ethyl acetate–methanol–water (1.5:5:2.75:5)
	11β,13-Dihydrolactucin Lactucin	Wu et al. (2007)	CCC/ethyl acetate–methanol–water (20:1:20)
	Peroxyferolide Lipiferolide	Graziose et al. (2011)	CPC/*n*-hexane–ethyl acetate–methanol–water (2:1:2:1)
	14-(3-Methylpentanoyl)-6-deoxybritannilactone 14-(3-Methylbutanoyl)-6-deoxybritannilactone 14-(2-Methylpropanoyl)-6-deoxybritannilactone 1,3-Epi-granilin 11,13-Dihydro-inuchinenolide B Pulchellin C 6-Deacetylbritanin 4H-Tomentosin Gaillardin Britannin	Fischedick et al. (2013a)	CPC/heptane–ethyl acetate–methanol–water (4:6:4:6)
	3β-Hydroxy-8β-[4′-hydroxytigloyloxy]-costunolide Eupalinolide A Eupalinolide B	Yan et al. (2012)	CCC/*n*-hexane–ethyl acetate–methanol–water (1:4:2:3)
	Xanthathin 4-Epi-xanthanol 4-Epi-isoxanthanol	Pinel et al. (2007)	CPC/*n*-hexane–ethyl acetate–methanol–water (1:1:1:1)
	β-Caryophyllene	Xie et al. (2008)	CCC/*n*-hexane–dichloromethane–acetonitrile (10:3:7)
	Rupestonic acid	Ma et al. (2005)	CCC/*n*-hexane–ethyl acetate–methanol–water (6:4:3.5:6.5) with 0.5 % acetic acid in stationary-phase
	Rupestonic acid	Yang et al. (2010)	CCC/*n*-hexane–ethyl acetate–methanol–water (3:5:3:5)
	Germacrone Curdione	Yan et al. (2005)	CCC/light petroleum ether–ethanol–diethyl ether–water (5:4:0.5:1)
	Atractylon Atractylenolide III	Zhao and He (2006)	CCC/light petroleum–ethyl acetate–ethanol–water (4:1:4:1)
	Nootkatone	Xie et al. (2009)	CCC/*n*-hexane–methanol–water (5:4:1)
	Caryophyllene oxide β-Farnesene Caryophyllene	Wei et al. (2012)	CCC/*n*-hexane–acetonitrile–ethanol (5:4:3)
	(*S*)-Dehydrovomifoliol	Yang et al. (2013)	CCC/*n*-hexane–ethyl acetate–methanol–water (1:5:1:5)
	Curdione Curcumol Germacrone Curzerene β-Elemene	Dang et al. (2010)	CPC/light petroleum ether–acetonitrile–acetone (4:3:1)
	Patchoulol	Li et al. (2011)	CPC/light petroleum ether–acetonitrile (1:1)
	Tussilagone 14-Acetoxy-7β-(3′-ethyl *cis*-crotonoyloxy)-lα-(2′-methyl butyryloxy)-notonipetranone 7aβ-(3′-Ethyl *cis*-crotonoyloxy)-lα-(2′-methyl butyryloxy)-3,14-dehydro-Z-notonipetranone	Wang et al. (2011a)	CCC/*n*-hexane–ethyl acetate–methanol–water (1:0.5:1.1:0.3)
	Blumenol C	Roscher and Winterhalter (1993)	CCC/chloroform–methanol–water (7:13:8)
	Solanesol	Hu et al. (2007)	CCC/*n*-hexane–methanol (10:7)
	Solanesol	Du et al. (2006)	CCC/light petroleum ether‐ethanol‐methanol (200:1:100)
	Kudtdiol	Rodrigues et al. (2009)	CCC/ethyl acetate–methanol–water (2:1.75:1)
	8ß-Hydroxyeremophil-3,7(11)-dien-12,8α 15,6α-Diolide and 8β-methoxyeremophil-3,7(11)-dien-12,8α;15,6α-diolide	Shi et al. (2008)	CCC/light petroleum–ethyl acetate–methanol–water (9:1:8:2)
Diterpenoids	Ptaerobliquol	Agostinho et al. (2013)	CPC/heptane–ethyl acetate–methanol–water) (6:5:6:5)
	Coniferin Coniferaldehyde glucoside	Slacanin et al. (1991)	CPC/chloroform–methanol–water (7:13:8)
	Salvinorin A	Shirota et al. (2007)	CPC/*n*-hexane–dichloromethane–methanol–water (8:8:9:2)
	Andrographolide Neoandrographolide	Du et al. (2003a)	CCC/*n*-hexane–ethyl acetate–methanol–water (1:4:2.5:2.5)
	Phytol	Xiao et al. (2013)	CCC/*n*-hexane–acetonitrile–methanol (5:5:3)
	Carnosol	Fischer et al. (1991)	CCC/*n*-hexane–ethyl acetate–methanol–water (70:30:14:8)
	15,16-Epoxy-12-hydroxy-8(17),13(16),14-labdatrien-19-oic acid Imbricatolic acid Isocupressic acid Sandaracopimaric acid Isopimaric acid	Martin et al. (2006)	CPC/chloroform–methanol–isopropanol–water (5:6:1:4)
	Kaurenoic acids Polyalthic acid	De Souza et al. (2010)	CCC/*n*-hexane–acetonitrile–ethyl acetate (1:1:0.4)
	Pseudolaric acid B *O*-β-d-glucopyranoside Pseudolaric acid C Deacetylpseudolaric acid A Pseudolaric acid A *O*-β-d-glucopyranoside Pseudolaric acid B Pseudolaric acid B methyl ester Pseudolaric acid A Pseudolaric acid H	He et al. (2012)	CCC/stepwise gradient: *n*-hexane–ethyl acetate–methanol–water (1:1:1:1) and (3:2:2:3) and (3.5:1:1:3.5)
	Oridonin	Lu et al. (2006)	CCC/*n*-hexane–ethyl acetate–methanol–water (1:2:1:2)
	Oridonin	He et al. (2011)	CCC/*n*-hexane–ethyl acetate–methanol–water (2.8:5:2.8:5)
	Oridonin Ponicidin	Lu et al. (2007)	2D-CCC/*n*-hexane–ethyl acetate–methanol–water (1:5:1:5) in 1st direction and (3:5:3:5) in 2nd direction
	Isoneotriptophenolide Hypolide Triptonide Triptophenolide Triptonoterpene methyl ether VI	Peng et al. (2008a)	CCC/*n*-hexane–ethyl acetate–methanol–water (3:2:3:2)
	Pseudolaric acids A and B And their glucosides (*O*-β-d-glucopyranosides)	Han et al. (2009)	CCC/*n*-hexane–ethyl acetate–methanol–water (5:5:5:5) for purification of aglicones and (1:9:4:6) for glycosides
	6β-Angeloyloxykolavenic acid 6β-Tigloyloxykolavenic acid	Wu et al. (2008)	CCC/*n*-hexane–ethanol–water (6:5:1)
	Pseudolaric acid B	Han et al. (2008)	CCC/*n*-hexane–ethyl acetate–methanol–water (5:5:5:5)
	lolitrem B	Grancher et al. (2004)	CPC/heptane–ethyl acetate–methanol–water (33:33:24:10)
	Carnosic acid Carnosol Carnosaldehyde Epirosmanol Rosmanol 12-Methoxy-carnosic acid Sageone	Fischedick et al. (2013b)	CPC/heptane–acetone–water (3:5:2)
	Stevioside Rebaudioside A Rebaudioside C	Huang et al. (2010)	CCC/*n*-hexane–*n*-butanol–water (1.5:3.5:5)
	Ginkgolides A Ginkgolides B Ginkgolides C Bilobalide	Liu et al. (2013b)	CCC/stepwise gradient: *n*-hexane–ethyl acetate–methanol–water (4:5:1:5) and (4:5:2:5)
	Cryptotanshinone Tanshinone I Tanshinone IIA	Gu et al. (2004, 2006)	CCC/stepwise gradient: *n*-hexane–ethanol–water (10:5.5:4.5) and (10:7:3)
	Cryptotanshinone Tanshinone I 1,2-Dihydrotanshinquinone Tanshinone IIA	Liang et al. (2013)	CCC/*n*-hexane–ethyl acetate–methanol–water (5:5:7:3)
	Tanshinone I Tanshinone IIA	Wu et al. (2010)	CCC/*n*-hexane–ethyl acetate–ethanol–water (8:2:7:3)
	Cryptotanshinone Tanshinone I Tanshinone IIA	Tian et al. (2000)	CCC/*n*-hexane–ethanol–water (4:2:2)
	Dihydrotanshinone Cryptotanshinone Methylenetanshiquinone Tanshinone I Tanshinone II Danshenxinkun B	Li and Chen (2001b)	CCC/stepwise gradient: *n*-hexane–ethanol–water (10:5.5:4.5) and (10:7:3)
	Tanshinone I Tanshinone IIA Dihydrotanshinone I Cryptotanshinone	Tian et al. (2002)	CCC/light petroleum–ethyl acetate–methanol–water (2:3:2.5:1.7)
	Przewaquinone A	Han et al. (2003)	CCC/carbon tetrachloride–methanol–water–*n*‐hexane (3:3:2:1)
	Triptolide	Ye et al. (2008b)	CCC/*n*-hexane–ethyl acetate–methanol–water (4:5:4:5)
	Dihydrotanshinone I 1,2,15,16-Tetrahydrotanshiquinone cryptotanshinone Tanshinone I Tanshinone IIA Neo-przewaquinone A Miltirone	Sun et al. (2011)	CCC/light petroleum–ethyl acetate–methanol–water (6:4:6.5:3.5)
Triterpenoids	2β,3β,4β-Trihydroxypregnan-16-one	Rodrigues et al. (2009)	CCC/*n*-hexane–ethyl acetate–methanol–water (1:2:1.75:1)
	Squalene	Lu et al. (2003)	CCC/*n*-hexane–methanol (2:1)
	Pristimerin Netzahualcoyene	Gutiérrez et al. (2007)	CPC/heptane–ethyl acetate–methanol–water (8:1:6:1)
	Guyanin	Severino et al. (2009)	CCC/*n*-hexane–ethanol–acetonitrile–water (10:8:1:1)
	Barbinervic acid Rotungenic acid 24-Hydroxy ursolic acid Ursolic acid	Fan and He (2006)	CCC/*n*-hexane–ethyl acetate–methanol–water (3:6:4:2)
	Betulinic acid	Frighetto et al. (2005)	CCC/*n*-hexane–ethyl acetate–methanol–water (10:5:2.5:1)
	Ursolic acid	Frighetto et al. (2008)	CCC/*n*-hexane–ethyl acetate–methanol–water (10:5:2.5:1)
	Bellericagenin B Bellericaside B Arjunglucoside I 28-Nor-17, 22-seco-2α, 3β, 19, 22, 23-pentahydroxy-Δ 12-Oleanane	Nasser et al. (2006)	CCC/chloroform–methanol–water (43:37:20)
	2α,3α,19β,23β-Tetrahydroxyurs-12-en-28-oic acid 2α,3α,23β-Trihydroxyurs-12-en-28-oic acid	Liu et al. (2011)	CCC/*n*-hexane–ethyl acetate–methanol–water (10:5:3:1)
	Oleanolic acid Ursolic acid	Du et al. (1995)	CCC/*n*-hexane–ethyl acetate–methanol–water (3:6:2:1)
	Shionone	Wang et al. (2012a)	CCC/*n*-hexane–methanol (2:1)
	Taraxasterol acetate Lupeol acetate β-Amyrin acetate	Abbott et al. (1989)	CCC/*n*-hexane–ethyl acetate–methanol–acetonitrile (5:2:4:5)
	Cycloartenyl ferulate 24-Methylene cycloartanyl ferulate	Liu et al. (2013a)	CCC/*n*-hexane–acetonitrile (1:1)
	Abrusoside A, B, C, D	Fullas et al. (1990)	CCC/chloroform–methanol–water (7:13:8)
	No compound names given	Marston et al. (1988)	CCC/chloroform–methanol–water (7:13:8)
	Inotodiol Trametenolic acid	Du et al. (2011)	CCC/*n*-hexane–ethyl acetate–methanol–water (1:0.4:1:0.4)
	Ursolic acid Ursolic acid lactone	Maurya and Srivastava (2011)	CPC/*n*-hexane–ethyl acetate–methanol–water (1:2:1.5:1) with 2 % ammonia solution in lower aqueous mobile phase (pH 9.5)
	Asiaticoside Madecassoside	Diallo et al. (1991)	CCC/chloroform–*n*-butanol–methanol–water (7:3:6:4)
	Taraxeryl acetate	Yang et al. (1995)	CCC/chloroform–methanol–water (2:2:1)
	Triterpenoic acid Acetyl-triterpenoic acid	Ito et al. (1990)	CCC/*n*-hexane–ethanol–water (6:5:2)
	Celastrol	Wu et al. (2004)	CCC/light petroleum–ethyl acetate–tetrachloromethane–methanol–water (1:1:8:6:1)
	Asiatic acid Madecassic acid Asciaticoside Madecassoside	Du et al. (2004)	CCC/*n*‐hexane–*n*‐butanol–0.05 M NaOH (5:1:6)
	Euscaphic acid Tormentic acid 2α-Acetyl tormentic acid 3β-Acetyl tormentic acid	Rocha et al. (2007)	CCC/gradient: *n*-hexane–ethyl acetate–methanol–water (1:2:1.25:2) and (1:2:1.5:2) and (1:2:1.75:2)
	Alisol B Alisol B 23-acetate	Yoon et al. (2009)	CPC/*n*-hexane–ethyl acetate–methanol–water (10:2:10:7)
	Ganoderic acids A, B, C6, D, E, F, G Ganoderenic acid D	Cheng et al. (2012)	CCC/stepwise gradient: light petroleum ether–ethyl acetate–methanol–water (3:5:3:5) and (4:5:4:5) chloroform–methanol–water (13:7:4) + ammonia (22 mM) in aqueous phase and TFA (11 mM) in organic phase for further purification
	Dehydrosulphurenic acid 3-Ketodehydrosulphurenic	Zhang et al. (2013a)	CCC/*n*-hexane–ethyl acetate–methanol–water (1:1.5:1.2:1)
	24-Methylene cycloartanol	Yao et al. (2007)	CCC/*n*-hexane–ethyl acetate–acetonitrile (5:1:4)
	Lupenone	Yao et al. (2007)	CCC/*n*-hexane–ethyl acetate–acetonitrile (5:2:5)
Triterpenoid saponins (ginsenosides)	Rc, Rb1 and Re	Wang et al. (2010)	CPC/ethyl acetate–*n*-butanol–water (1:1:2)
	Rg3, Rk1, Rg5 and F4	Ha et al. (2007)	CCC/methylene chloride–methanol–water–isopropanol (6:6:4:1)
	Rf, Rd, Re and Rb1	Qi et al. (2010)	CCC/methylene chloride–methanol–5 mM aqueous ammonium acetate–isopropanol (6:2:4:3)
	Re	Engelberth et al. (2010)	CPC/heptane–n-butanol–water (3:4:7)
	Rg1, Re, Rf, Rh1, Rb1, Rc, Rb2 and Rd	Shehzad et al. (2011)	CCC/chloroform–methanol–water–isopropanol (4:3:2:1)
	Re,Rb1, Rc and Rb2	Cheng et al. (2011)	CCC/methylene chloride–methanol–water–isopropanol (6:2:4:3, v/v) further purification of Rb1, Rc and Rb2 in *n*-hexane–*n*-butanol–0.1 % formic acid (0.7:3:4)
	Rb1, Rb2, Rc, Rd, Re, Rg1, Rf and Rh 1	Shehzad et al. (2012)	CCC/stepwise gradient: *n*-hexane–ethyl acetate–methanol–water (5:6:1:4) and (4:3:1:2) and (3:3:1:2)
	Re and Rg1	Chen et al. (2012)	CCC/ethyl acetate–*n*-butanol–water (4:1:6)
	Rh1, Rf, Rd, Rg1, Re, Rc, Rb2 and Rb1	Shehzad et al. (2013)	CCC/methylene chloride–methanol–water–isopropanol (1:1:2:1)
	Rg6, Rg5, Rk1,F4, Rg3, Rg2, Rf, Rd, Rg1, Re, Rc, Rb2, Rb1	Shehzad et al. (2013)	CCC/stepwise gradient: methylene chloride–methanol–water–isopropanol (5:4:1:3) and (2:2:1:2)
	Rb1 and Rb2	Wang et al. (2013)	CPC/ethyl acetate–*n*–butanol–water (0.8:1.2:2)
	Rg, Rd, Re and Rb Notoginsenoside R	Cao et al. (2003)	CCC/chloroform–*2*-butanol–methanol–water (5:1:6:4)
	Rg, Rd, Re and Rb Notoginsenoside R	Cao et al. (2003)	CCC/ethyl acetate–*n*–butanol–water (1:1:2)
	Rg, Re and Rb Notoginsenoside R	Du et al. (2003b)	CCC/*n*-hexane–*n*-butanol–water (3:4:7)
	Ro	Cheng et al. (2010)	CCC/ethyl acetate–isopropanol–0.1 % formic acid (3:1:5)
	Rg1, Re and Rb1 Notoginsenoside R1	Wang et al. (2011b)	CPC/ethyl acetate–*n*-butanol–water (1:1:2)
Triterpenoid saponins	Lucyoside Q Lucyoside H	Du and Gao (2006)	CCC/chloroform‐methanol‐water (13:7:8)
	Astragaloside I Astragaloside II	Han et al. (2007)	CCC/stepwise gradient: ethyl acetate–*2*-propanol–water (5:1:5) and (50:1:50)
	Lancemaside A Foetidissimoside A Astersaponin Hb	Shirota et al. (2008)	CPC/*n*-hexane–*n*-butanol–methanol–0.1 % aqueous formic acid (3:4:1:6)
	Saikosaponins-A Saikosaponins-C	Yoon and Kim (2009)	CPC/ethyl acetate–*n*-butanol–methanol–water (15:1:3:1)
	Platycoside E Deapio-platycoside E	Han et al. (2009)	CCC/*n*-hexane–*n*-butanol–water (1:40:20)
	Platycodin D3 Deapio-platycodin D3 Platycodin D Deapio-platycodin D	Han et al. (2009)	CCC/*n*-hexane–*n*-butanol–water (1:10:5)
	2″-*O*-Acetylplatycodin D 3″-*O*-Acetylpolygalacin D 2″-*O*-Acetylpolygalacin 3″-*O*-Acetylplatycodin D Polygalacin D	Ha et al. (2011)	CCC/chloroform–methanol–isopropanol–water (3:2:2:3)
	No compound names given	Shi et al. (2007)	CCC/ethyl acetate–*n*-butanol–ethanol–0.05 % TFA (5:10:2:20)
	Astragaloside IV Astragaloside II Astragaloside I Acetylastragaloside I	Peng et al. (2008b)	CCC/stepwise gradient: *n*-hexane–ethyl acetate–ethanol–water (1:0.6:0.6:1) and (1:1:1:1)
	Gypsogenin derivatives Dianoside C	Yao et al. (2008)	CCC/*n*-hexane–*n*-butanol–methanol–0.02 % TFA (1:9:1:9)
	Hederagenin 3-*O*-[β-d-xylopyranosyl-(1→3)-α-l-rhamnopyranosyl-(1→2)-l-arabinopyranosyl]-hederagenin	Xin et al. (2009)	CCC/*n*-hexane–ethyl acetate–methanol–water (7:3:5:5)
	23-*O*-Acetylshengmanol-3*O*-d-xylopyranoside Cimiracemoside D 25-*O*-acetylcimigenol-3-*O*-d-Xylopyranoside Cimigenol	Cicek et al. (2010)	CCC/*n*‐hexane–acetone–ethyl acetate–isopropanol–ethanol–water (3.5:1:2:1:0.5:2)
	Esculentosides A, B, C, and D	Ma et al. (2010)	CCC/chloroform–methanol–water (4:4:2)
	Hederagenin		CCC/heptane–acetone–methanol (5:1:4)
	Hederagenin	He et al. (2002)	CPC/*n*-hexane–ethyl acetate–methanol–water (7:8:5:3)
	Elatoside F	Lee et al. (2009)	CCC/chloroform–methanol–water–isopropanol (4:3:3:1)
	Tormentic acid	Wang et al. (2012b)	CCC/*n*-hexane–ethyl acetate–methanol–water (4:5:4:5)
	Arganine A, C, D Tieghemelin	Gosse et al. (2002)	CCC/*tert*-butylmethyl ether–*n*-butanol–acetonitrile–0.5 % TFA (1:3:3:5)
	Triacetyl soyasaponin Ab, Aa, Ab, Ae, Ba, Af, Bb, Be and conjugated groups, αg, βg and γg	Zhao et al. (2012)	CCC/*n*-butanol–acetic acid–water (5:0.05:5)-acid in *n*-butanol as stationary phase
	Licorice-saponin A3 Glycyrrhizic acid, 3-*O*-[β-d-glucuronopyranosyl-(1→2)-β-d-Galactopyranosyl]glycyrrhetic acid	Xu et al. (2013)	CCC/ethyl acetate–*n*-butanol–water (2:3:5) with 10 mM TFA in the upper organic Stationary phase and 10 mM ammonia in the lower aqueous mobile phase
	Glycyrrhizin	Jiang et al. (2004)	CCC/ethyl acetate–methanol–water (5:2:5)
	Goyaglycoside-E Momordicoside L Goyaglycoside-a Momordicoside K	Du and Yuan (2005)	CCC/*tert*-butylmethyl ether–*n*-butanol–methanol–water (1:2:1:5) or (1:3:1:5)
Tetranortriterpenoids	Azadirachtin A Azadirachtin B Azadirachtin H Desacetylnimbin Desacetylsalannin Nimbin Salannin	Silva et al. (2007)	CCC/*n*-hexane–*n*-butanol–methanol–water (1:0.9:1:0.9)
	Methyl angolensate 7-Descetoxy-7-oxogedunin Deacetylgedunin 6α-Acetoxygedunin Gedunin Andirobin	da Silva et al. (2009)	CCC/stepwise gradient: *n*-hexane–ethyl acetate–methanol–water (2:1:1.5:1) and (2:1:1.75:1)
Tetraterpenoids (Carotenoids)	Cochloxanthin Dihydrocochloxanthin	Diallo and Vanhaelen (1988)	CCC/tetrachloromethane–methanol–water (5:4:1)
	Zeaxanthin	Chen et al. (2005)	CCC/*n*-hexane–ethyl acetate–ethanol–water (8:2:7:3)
	Crocin	Jiang et al. (2011)	CCC/*tert*-butylmethyl ether–*n*-butanol–acetonitrile–water (2:2.5:1:5)
	Fucoxanthin	Kim et al. (2011)	CPC/*n*-hexane–ethyl acetate–ethanol–water (5:5:7:3)
	Lutein	Wei et al. (2003)	CCC/*n*‐heptane–chloroform–acetonitrile (10:3:7)
	Zeaxanthin Lutein	Aman et al. (2005)	CCC/*n*-hexane–ethanol–water (6:5:1.3)
	Lutein	Tsao and Yang (2006)	CCC/*n*-hexane–ethanol–water (6:4.5:1.5)
	Canthaxanthin	Li et al. (2006)	CCC/*n*-hexane–ethanol–water (10:9:1)
	9′-*Cis*-neoxanthin	Baldermann et al. (2007)	CCC/*n*-hexane–ethanol–water (5:5:4.5)
	Lycopene	Baldermann et al. (2008)	CCC/*n*-hexane–dichloromethane–acetonitrile (30:11:18) with 85 mg/L of 3-tert-butyl-4-hydroxyanisol and 2-tert-butyl-p-cresol
	Crocins 1, 2, 5 Picrocrocin	Lechtenberg et al. (2008)	CPC/*n*-hexane–ethyl acetate–ethanol–water (1:3:4:7)
	Fucoxanthin	Xiao et al. (2012)	CCC/*n*-hexane–ethyl acetate–ethanol–water (5:5:6:4)
	Lutein	Li et al. (2001)	CCC/*n*-hexane–ethanol–water (4:3:1)
	Lycopene	Wei et al. (2001)	CCC/*n*-hexane–dichloromethane–acetonitrile (10:3.5:6.5)
	Astaxanthin	Li and Chen (2001a)	CCC/*n*-hexane–ethyl acetate–ethanol–water (5:5:6.5:3)
	Tephrosin Deguelin 6a,12a-Dehydrodeguelin	Ye et al. (2008a)	CCC/*n*-hexane–ethyl acetate–methanol–water (1:0.8:1:0.6)

*CCC* counter-current chromatography, *CPC* centrifugal partition chromatography

The solvent systems tables mentioned above are presented here, sorted according to class of terpenoid separated. That table is presented for the benefit of CCC users by suggesting possible suitable solvent systems, which can act as a starting point for further refinement and optimization in the composition of the system.

## Applications of high speed and high performance CCC

Being a liquid–liquid chromatography system, CCC can select from an almost infinite range of possible two-phase solvent systems for a purification. Most reported purifications with the technique have understandably concentrated on compounds of intermediate polarity. For example, a review of Chinese herbal medicines purified by CCC found a total of 214 different compounds in 198 published papers with a LogP polarity range from −4 to +12 (Sutherland and Fisher 2009). However, more than 60 % of those compounds fell in the narrow intermediate polarity range of 0–4. Nevertheless, CCC and its sister technique, CPC can be particularly useful for purifications in the extreme polar and non-polar range and some impressive examples have been published. In 1995, Gasper and co-workers managed to purify C_60_ and C_70_ fullerenes using the non-polar and non-aqueous solvent system of isooctane, dimethylformamide, 1,2-dichlorobenzene (4:2:1) plus 1 % *tert*-butylmethyl ether (Gasper et al. 1995). Still at the non-polar end of the spectrum, the carotenoid lycopene was isolated from tomato paste using a non-aqueous phase system of *n*-hexane, dichloromethane and acetonitrile (10:3.5:6.5) (Wei et al. 2001). This separation was performed in a single step from the crude material with 100 mg of crude extract injected onto a 230 ml capacity centrifuge. The purity of the final product was measured as >98 % by HPLC peak area. Lycopene has a calculated LogP value of approximately 17.6. Beyond the carotenoids, non-polar terpenoids can also be challenging to purify with solid phase chromatography, and again CCC offers an alternative approach. In one example, Liu et al. (2013a) successfully separated cycloartenyl ferulate and 24-methylene cycloartanyl ferulate. Due to the weak polarity, a series of low-polar solvent systems, including: *n*-hexane–ethyl acetate–ethanol (methanol)–water, *n*-hexane–ethanol (methanol)–water, *n*-hexane–methanol and *n*-hexane–ethyl acetate–*n*-butanol ethanol (methanol)–water were tested, but all without success. Since the HPLC mobile phase for the analysis of those two target compounds consisted of methanol, acetonitrile and isopropanol, these three solvents were further studied and a mixture of hexane and acetonitrile (1:1) was used for the purification. Another application of non-aqueous solvent systems is the purification of shionone—also a low-polar compound. Wang et al. (2012a) tested several hydrophobic two-phase solvent systems. Among these *n*-hexane–methanol (2:1) and heptane–dichloromethane–acetonitrile (20:7:13) were suitable for the separation.

Turning to the extreme polar end of the spectrum, CCC has been used with aqueous-organic solvent systems for the purification of peptides. For example, the peptide antibiotic colistin (LogP about −4.7) was isolated from a commercial microbial preparation using the polar two-phase system consisting of *n*-butanol and 0.04 M aqueous trifluoroacetic acid (1:1) (Ikai et al. 1998). Using the salt-based solvent system consisting of 1-propanol, acetonitrile, saturated ammonium sulphate and water (1:0.5:1.2:1) the highly polar glucosinolate glucoraphanin was purified to a purity >98 % from a crude broccoli extract in a single step (Fisher et al. 2005).

Sometimes modifications are necessary. The separation of three closely related triterpenes: sericic acid, trachelosperogenin E and sericoside was achieved using a three-phase solvent system composed of *n*-heptane, *tert*-butylmethyl ether, acetonitrile and water. After mixing all solvents in equal volume, the upper phase was separated and *tert*-butylmethyl ether was shaken with the remaining two-phase solvent system in order to decrease the polarity. The lower phase was used as a stationary phase. Sericic acid was identified in the fractions collected when the upper phase was used as the mobile phase, while the elution of the middle phase led to the separation of trachelosperogenin E and sericoside (Hamzaoui et al. 2013).

Zhao and Du (2007) proposed a novel non-aqueous two-phase solvent system composed of sunflower oil and ethanol to separate solanesol—a non-cyclic terpene alcohol used as a food additive for preventing cardiac arrest and cancer. Before, this target molecule was purified from tobacco leaves extract with petroleum and methanol, both solvents being unsuitable for the food industry.

Sometimes the addition of a small amount of acid can significantly improve the purification. Shirota et al. (2008) purified the saponins lancemaside A, foetidissimoside A and astersaponin Hb from a hot water extract of *Codonopsis lanceolata* roots used CPC with mixture of *n*-hexane–*n*-butanol–methanol–0.1 % aqueous formic acid (3:4:1:6) as the two-phase solvent system. Because the crude extract contained large amounts of sugars, an aqueous phase was chosen as the stationary phase so that these sugars would be retained in the partition cells, whereas an organic phase was chosen as mobile phase for elution of the target compound. Addition of formic acid to the two-phase solvent system retained the carboxy group in the non-ionised form and allowed the separation of the lancemasides from the large amounts of water-soluble sugars present. The addition of acetic acid to the ethyl acetate–*n*-butanol–water mixture improved the resolution of soyasaponins, naturally occurring triterpenoid glycosides. A suitable mixture *n*-butanol, acetic acid, water (5:0.05:5) was taken but acetic acid was added only in *n*-butanol as the stationary phase (Zhao et al. 2012). During the separation, with the movement of the mobile phase the acetic acid was gradually diluted with water and the polarity of the stationary phase changed as a gradient. It is worth mentioning that the main difficulties with soyasaponin isolation and purification are that the soyasaponins coexist in soybeans with the isoflavone glycosides and they share overlapping polarities. There is also the structural similarity of soyasaponin compounds.

CCC is popularly applied for purification of ginsenosides, an important group of terpenoids due to their biological activity. They generally fall in the moderate to polar category and therefore require similar solvent systems for purification (Qi et al. 2010; Shehzad et al. 2013). A good solubility for saponin-like compounds can be provided by the addition of chloroform or other chlorinated solvents and a gradual polarity change between phases can be achieved by varying the methanol ratio in the system or by adding another alcohol like isopropanol (Qi et al. 2010). Shehzad et al. (2013) proposed an efficient CCC separation method in which a flow-rate gradient technique was coupled with a new solvent gradient dilution strategy for the isolation of ginsenosides from Korean red ginseng (steam-treated *P. ginseng*). The column was first entirely filled with the upper stationary phase mixture of methylene chloride–methanol–isopropanol–water (5:4:1:3) in a reversed phase system. After 300 min, when five ginsenosides had eluted, the flow rate was increased from 1 to 1.2 ml/min and also the dilution of the lower phase was initiated and was changed to 100 % of lower phase composed of the mixture mentioned above in a ratio (2:2:1:2). Overall, 13 ginsenosides including Rg1, Re, Rf, Rg2, Rb1, Rb2, Rc, Rd, Rg3, Rk1, Rg5, Rg6, and F4 were purified (Shehzad et al. 2013). Qi et al. (2010) for the purification of ginsenosides Rf, Rd, Re and Rb1, applied mixtures of methylene chloride, methanol, water and isopropanol (6:2:4:3).

Emulsification can often be a problem in the CCC purification of ginsenosides. Generally, adding an electrolyte, such as salt or acid, can help to eliminate such emulsification. Salt is not recommended because an additional desalting process will be necessary after the separation. Therefore, formic acid is often chosen to prevent emulsification (Cheng et al. 2010). However, Qi et al. (2010) preferred to avoid an acidic environment, which would lead to the decomposition of ginsenosides. Instead, the inorganic salt ammonium acetate was chosen, because it is volatile and can be precipitated in warm acetone for sample recovery. The addition of this salt resulted in a very slight decrease in *K* values and thus a shorter separation time. In the end, the proposed solvent system was methylene chloride–methanol–5 mg/mL aqueous ammonium acetate–isopropanol (6:2:4:3).

As the solvent system contained methylene chloride and methanol, both toxic to humans and the environment, Wang et al. (2010) chose low toxic solvents such as ethyl acetate and *n*-butanol. Since the ginsenosides in *P. quinquefolium* L. have a comparatively larger polarity with solubility in hydrophilic solvents, both phases require a certain hydrophilicity to get a good separation. Initially, the hexane–*n*-butanol–water solvent systems were scanned at several volume ratios, but all the systems had a poor retention in the CPC column. Then ethyl acetate was then employed instead of hexane in order to enhance the retention of the stationary phase in the column. Because ginsenosides are easily dissolved in *n*-butanol, and the viscosity of *n*-butanol is comparatively large, so in an ethyl acetate–*n*-butanol–water solvent system, the addition of *n*-butanol could delay the peak elution time and affect the separation. Overall, the system of ethyl acetate–*n*-butanol–water (1:1:2) was used for successful separation of three ginsenosides Rc, Rb1, and Re.

Counter-current chromatography has also been used for the separation of minor and structurally similar compounds. Fan and He (2006) using HSCCC with *n*-hexane–ethyl acetate–methanol–water (3:6:4:2) to separate not only ursolic acid, which is a very small content of the leaves of *Diospyros kaki*, but also two other pentacyclic triterpenes: barbinervic acid and its epimer rotungenic acid, differing only with the configuration of a hydroxyl group at position C3: one contains an axial and the other an equatorial hydroxyl group. HSCCC was also employed for the separation and purification of minor constituents in *Platycodi Radix*. Platycosides, the saponins that are the major active constituents, are typically composed of oleanene backbones with two side chains; one a glucose unit attached through an ether linkage at the C-3 position of a triterpene, and the other 28-*O*-arabinoserhamnose-xylose-apiose linked by an ester bond. They also have different substituents at the C-4 position. Because the content of these compounds is very low, conventional methods are frequently not suitable for the separation. Ha et al. (2011) used a two-phase solvent system consisting of chloroform–methanol–isopropanol–water (3:2:2:3) for purification of minor saponins 2″-*O*-acetylplatycodin D, 3″-*O*-acetylpolygalacin D, 2″-*O*-acetylpolygalacin and a mixture of 3″-*O*-acetylplatycodin D and polygalacin D, which were further successful purified using prep-HPLC (Ha et al. 2011). Ha and Kim (2009) also used HSCCC for the separation of three pairs of platycosides and their deapiose forms. They used an interesting modification: the column was first filled with a mixture of the two phases, thus reducing the amount of time for hydrodynamic equilibrium to be established. The ratio of two phases was optimised at 70:30 (stationary phase–mobile phase) based on the amount of time required to reach hydrodynamic equilibrium. A series of solvent systems were tested. In the gradient elution mode, the retention of the stationary phase was extremely low. The authors decided to perform the separation in two stages. First, platycoside E, deapio-platycoside E, a mixed fraction containing platyodin D and deapiose form, and a fraction containing platycodin D3 and deapiose form were separated using the *n*-hexane–butanol–water (1:40:20) in reversed-mode. Then mixed fractions I and II were further purified in the normal elution mode with the above mentioned solvent system in the ratio 1:10:5.

CCC/CPC seems to be one of the few efficient approaches for the separation of xanthanolides, a bicyclic subtype of sesquiterpenic lactones characterized by a 5,7-fused system containing a γ-lactone moiety. Because of some well documented and promising activity purification of these compounds is important. The presence of several chiral centres probably explain the difficulty obtaining enantiomericaly pure xanthanolides by total synthesis, thus purification from natural source seems to be the best solution. Xanthonolides are coextracted with chlorophyll and lipids. The pigment crystallization and the delipidation in order to clean the extract is not selective and cause the partial loss of target compounds. Purification of several xanthanolides (xanthathin, 4-*epi*-xanthanol and 4-*epi*-isoxanthanol) was realized in one step, directly from the crude chloroformic extract of the leaves of *X. macrocarpum* with a mixture of heptane–ethyl acetate–methanol–water (1:1:1:1) (Pinel et al. 2007).

High-speed counter-current chromatography can be applied as a method suitable for fingerprinting. Gu et al. (2006) used it for quality control of TCMs and identification of the active compounds of *Salvia miltiorrhiza* Bunge, a popular traditional Chinese medicine. In order to purify a series of tanshinones a stepwise elution with solvent systems composed of *n*-hexane–ethanol–water (10:5.5:4.5) and (10:7:3) was used. The method was compared with more conventional approaches, such as high performance liquid chromatography (HPLC), high performance capillary electrophoresis (HPCE), and thin-layer chromatography scan (TLCS). In the HSCCC separation, 12 components were separated, with good resolution and precision, within 13 h. HSCCC showed better performance in the analysis of tanshinones, which produced a fingerprint which contained more chemical information than that of e.g. TLCS.

Lu et al. (2007) proposed this effective two-dimensional counter-current chromatographic method for the simultaneous isolation and purification of oridonin and ponicidin from a crude extract of *Rabdosia rubescens* with a pair of two-phase solvent systems composed of *n*-hexane–ethyl acetate–methanol–water (1:5:1:5 and 3:5:3:5, v/v). A combination of stepwise CCC and pH-zone-refining is also possible. Cheng et al. (2012) reported its successful combination in the separation of the main components from *G. lucidum*. In the first step, a two-phase solvent system composed of petroleum ether–ethyl acetate–methanol–water (3:5:3:5 and 4:5:4:5) led to the separation of ganoderic acids GE, GC6 and GF with high purity in one run. Also two peaks containing GG, GB, GA and GED, GD, respectively, were collected and their separation was followed by pH-zone-refining CCC. Chloroform–methanol–water (13:7:4) with NH_4_OH in upper aqueous stationary phase and trifluoroacetic acid as the eluter acid was the most suitable. In another example of two-dimensional CCC, a three-step gradient elution and two-step flow-rate gradient elution was applied to separate 8 diterpene compounds within 80 min in a single run from the alcohol extract of *Pseudolarix kaempferi*. Pseudolaric acid B *O*-β-d-glucopyranoside, pseudolaric acid C, deacetylpseudolaric acid A, pseudolaric acid A *O*-β-d-glucopyranoside, pseudolaric acid B, pseudolaric acid B methyl ester, pseudolaric acid A and pseudolaric acid H were obtained with very high purity (He et al. 2012). The separation was performed in normal phase system. In the first step, the mobile phase, composed of hexane–ethyl acetate–methanol–water (1:1:1:1), was used with a flow rate 0.5 ml/min. While the mobile phase (upper) of (2:3:2:3) and (1:3.5:1:3.5) were used in the second and third step, and the flow rate of mobile phase was set at 1 ml/min. When the separation was performed in preparative conditions, the initial flow rate of mobile phase was 25 ml/min in the first step and then increased to 50 ml/min in the second and third step.

## Principle advantages and drawbacks of CCC in the purification of natural products

Counter-current chromatography has a number of key advantages in the purification of natural products. For example, the solvent usage is generally far lower than that of solid phase chromatography systems operating at the same scale (about 25 %) (Graham et al. 2001). Furthermore, since the process is frequently an isocratic one, a simple analysis of solvent composition allows the recycling of the solvents, reducing the usage still further (Garrard et al. 2007). The technique also allows for 100 % recovery of the sample components. In other words, the target compound can always be retrieved since there is no solid phase and therefore no possibility of losses arising from irreversible adsorption onto the solid matrix. This is a significant advantage in every purification process. As a solid-free and therefore relatively gentle chromatographic technique, CCC can be used for the isolation of unstable natural compounds. Baldermann et al. (2007) presented the successful purification of 9′-*cis*-neoxanthin, the predominant isomer of neoxanthin in green vegetables. Major problems during its isolation include isomerization and oxidation, mainly caused by higher temperatures, light or oxygen exposure. When typical solid stationary phase techniques are applied, the rearrangement products can be detected or complete isomerization can be observed. A solid-free technique is a very practical solution for avoiding the above mentioned complications.

Particulates, such as cell debris, are generally well tolerated in CCC, particularly when performed at large scale where the tubing bore may be up to 10 mm in diameter. This is another major advantage over solid phase chromatography systems. Thus filtering a sample is frequently not necessary, depending on the scale of CCC employed, and even if it is required, it is usually a simple filter paper filtration. With processing times similar to that of other purification methods, scale up is also possible with modern instruments with examples existing running from milligram to kilogram levels (Garrard et al. 2008). The technique can be operated in normal batch injection mode, or as a continuous extraction process for better throughput. A wide range of polarities can be processed due to the range of solvents that may be used (the literature reports examples with a logP range from −4.7 (colistin peptide antibiotic) (Ikai et al. 1998) to +17.6 (lycopene) (Wei et al. 2001). Also, the separation of compounds with vastly different polarities from a single extract is possible. For example, in order to purify a wide range of polarity of triterpene saponis from *Panax notoginseng*, Zhang et al. (2013b) successfully coupled accelerated solvent extraction (ASE) and high-performance counter-current chromatography (HPCCC). First the upper phase of the solvent system ethyl acetate–*n*-butanol–water (1:1:2 or 1.2:1:2) or ethyl acetate–*n*-butanol–methanol–water (3:5:1.5:6) was used as both the ASE solvent and HPCCC stationary phase. The polar saponis were eluted. In order to separate fractions with moderate polarity, the upper phase of system ethyl acetate–*n*-butanol–methanol–water (6:3:2:6 or 7:3:2:7) was used. Finally, the upper phase of the solvent system of *n*-hexane–*n*-butanol–methanol–water (8:2:2:8) or *n*-hexane–ethyl acetate–*n*-butanol–methanol–water (0.2:10:0.5:1.5:8) was used as both the ASE solvent and HPCCC stationary phase to elute the low polar compounds. This combination of methods allowed the purification of notoginsenosides R6, R1, Spt1 and ginsenosides Rb1, F4, Rh3, Rg3, Rs3 and Rk1 with a wide range of polarity (Zhang et al. 2013b).

Compared to the early instruments, the quality of modern CCC apparatus is very good. In most cases the coils are tough and the machinery robust. A set of coils would be expected to last the lifetime of the centrifuge and maintenance and running costs are low. Unlike solid phase chromatography, there is no change to component retention over time (no column aging effects) as a freshly-filled coil of solvents is used each run. This makes it easier to consistently satisfy current regulatory requirements when performing purifications under a good laboratory practice (GLP) or good manufacturing practice (GMP) environment.

As has been seen in a number of quoted examples, a large advantage of CCC is that the technique can be operated in a number of different modes, since both the mobile and the stationary phase are liquid. Either normal or reverse phase chromatography can be chosen, depending on which solvent phase is selected to be the mobile one. However, it is even possible to switch from normal phase elution to reverse phase (or vice versa) in the middle of a run. Intermittent counter-current extraction (ICCE) is a continuous process where the operation alternates between normal and reverse phase mode at regular intervals, with the sample continuously introduced in the middle of the coil (Hewitson et al. 2009). In the reference quoted, this technique was successfully used to purify a diterpenoid, triptolide, a high value target compound, from a Chinese herbal plant, from 2 % in the crude extract to over 98 % purity. This was achieved by retaining and enriching the target compound within the CCC coil while washing away all the other components of the crude material. Alternatively, components can be recovered by eluting the liquid stationary phase without any compound losses whilst maintaining resolution (Berthod et al. 2003), a technique known as elution-extrusion. This technique is frequently adopted at the end of standard CCC purification runs simply to ensure that no loss of target compound has occurred within the liquid stationary phase. Another possibility is co-current chromatography (Berthod and Hassoun 2006) where both phases are pumped in the same direction. All of these options use a conventional CCC centrifuge and are thus easy to implement in the laboratory or pilot plant. However, with modifications to the CCC coils, continuous counter-current extraction (CCCE) is possible, where one phase is pumped in the opposite direction to the other and the sample is introduced continuously into the centre of the coil (Ito et al. 2006; Van den Heuvel 2007).

The method also allows a two dimensional procedure to be applied. The purification of very similar terpene lactones from *G. biloba* L, such as bilobalide, ginkgolides A, B, C, and J, is an example. The partitioning experiment assisted the design of a 2D procedure using a pair of orthogonal solvent systems: chloroform–methanol–water (10:7:3) and hexane–ethyl acetate–methanol–water (4:6:4:6) with addition of 0.5 % DMSO to increase the resolution of ginkgolides A and B. This approach separated these almost equipolar lactones (Qiu et al. 2012).

The main drawbacks of the technique, particularly when compared to preparative HPLC and other solid phase chromatography techniques, include a lower efficiency. When measured in terms of theoretical plates, the efficiency of a “good” CCC apparatus is in the low thousand plate range. This figure cannot be directly compared to HPLC plate counts due to the much higher percentage of stationary phase (around 80 % compared to perhaps 5 % active stationary phase sites in HPLC) and the resolution of CCC can be extremely good. However, a low efficiency results in broad peaks, making the technique far more suited to a preparative application as opposed to an analytical one. Also the narrow polarity range within each run should be emphasised. As mentioned above, a wide range of polarities can be processed by CCC by using different solvent systems. However, a single CCC run operates over a relatively narrow polarity window. Although some examples of gradient elution have been reported (see Table 2), these do not give as wide a polarity range as that achievable with HPLC. The narrow polarity window can be used to advantage when it is desired to pluck a single target compound from a complex mixture. However, it is a disadvantage when a dozen pure compounds are required from the mixture in a single purification run. The CCC apparatus does not inherently lend itself to easy automation and thus its operation can appear labour-intensive. In addition, the instruments have not received the same intensive commercial development that has made modern HPLC and GC equipment so sophisticated. This situation will undoubtedly improve in the future, with advances in CCC machine design. Finally, the solvent system selection is undoubtedly time-consuming. As the two phases are liquid, changes in one phase directly affects the other. Analysis of a range of possible solvent systems can be performed by an automated liquid-handling robot but this still requires a number of hours to complete. This is a current area of research in the field and no doubt large improvements in solvent system selection time can be expected over the next few years.

## Conclusions

Although initially dismissed by many chemists and purification scientists as slow, unreliable and temperamental, steady development of the technique of CCC on both the engineering and the application side has transformed it into a technique worthy of inclusion in the natural product scientist’s arsenal. On one side, engineering developments have produced machines that are robust, capable of fast, efficient separations and able to accept high injection loadings. On the other side, developments in the application protocols have produced modes of operation and solvent systems to purify out compounds from the full polarity spectrum. Combined, these have produced a technique that is wonderfully suited to natural product purifications, particularly on a large preparative scale, with advantages over solid phase techniques such as the ability to accept particulates and to always recover all components, and advantages over the old-style liquid–liquid techniques such as high speed, high loading and high resolution. To assist users of CCC and those wishing to experiment with the technique, a comprehensive phase selection table has been generated by distilling the solvent system information presented from CCC terpenoids purifications over the last 30 years.
